# Tracking of *Borrelia afzelii* Transmission from Infected *Ixodes ricinus* Nymphs to Mice

**DOI:** 10.1128/IAI.00896-18

**Published:** 2019-05-21

**Authors:** Tereza Pospisilova, Veronika Urbanova, Ondrej Hes, Petr Kopacek, Ondrej Hajdusek, Radek Sima

**Affiliations:** a Institute of Parasitology, Biology Centre of the Czech Academy of Sciences, Ceske Budejovice, Czech Republic; b Faculty of Science, University of South Bohemia, Ceske Budejovice, Czech Republic; c Department of Pathology, Charles University, Medical Faculty and Charles University Hospital, Plzen, Czech Republic; Yale University School of Medicine

**Keywords:** *Borrelia*, *Borrelia afzelii*, *Ixodes ricinus*, Lyme disease, tick-borne pathogens, transmission

## Abstract

Quantitative and microscopic tracking of Borrelia afzelii transmission from infected Ixodes ricinus nymphs has shown a transmission cycle different from that of Borrelia burgdorferi and Ixodes scapularis. Borrelia afzelii organisms are abundant in the guts of unfed I. ricinus nymphs, and their numbers continuously decrease during feeding. Borrelia afzelii spirochetes are present in murine skin within 1 day of tick attachment. In contrast, spirochetes were not detectable in salivary glands at any stage of tick feeding. Further experiments demonstrated that tick saliva is not essential for B. afzelii infectivity, the most important requirement for successful host colonization being a change in expression of outer surface proteins that occurs in the tick gut during feeding. Spirochetes in vertebrate mode are then able to survive within the host even in the absence of tick saliva. Taken together, our data suggest that the tick gut is the decisive organ that determines the competence of I. ricinus to vector B. afzelii. We discuss possible transmission mechanisms of B. afzelii spirochetes that should be further tested in order to design effective preventive and therapeutic strategies against Lyme disease.

## Abstract (precis)

Quantitative and microscopic tracking of Borrelia afzelii transmission from infected Ixodes ricinus nymphs has shown a transmission cycle different from that of Borrelia burgdorferi and Ixodes scapularis. Borrelia afzelii organisms are abundant in the guts of unfed I. ricinus nymphs, and their numbers continuously decrease during feeding.

## INTRODUCTION

Lyme borreliosis is the most common vector-borne disease in Europe and the United States. It is caused by the spirochetes Borrelia burgdorferi in the United States or by the B. burgdorferi *sensu lato* complex, comprised of B. afzelii, B. garinii, and B. burgdorferi, in Europe. *Borrelia* spirochetes are maintained in nature through an enzootic cycle involving small vertebrates, primarily rodents and birds, and are vectored by ticks of the genus *Ixodes* (1).

Understanding the complex interactions within the tick-*Borrelia*-host triangle is indispensable for the development of efficient vaccines or drugs against Lyme disease. Progress in understanding borreliosis transmission has been achieved during the last 3 decades, mainly in the United States, by investigation of B. burgdorferi strains vectored by Ixodes scapularis. Three hypotheses for *Borrelia* transmission were proposed in earlier studies. The first hypothesis favored a direct infection via mouth parts by regurgitation of the spirochetes present in the midgut contents (2). The second suggested a salivary route of transmission that assumed systemic distribution of spirochetes within the tick body (3). In the third hypothesis, infection via contaminated feces was also considered (2, 4) but was soon abandoned (5). A number of ensuing studies corroborated the salivary route of B. burgdorferi transmission by I. scapularis as the most likely. Therefore, the current, generally accepted model of Lyme disease transmission can be summarized as follows. At the beginning, larval I. scapularis organisms acquire *Borrelia* spirochetes from infected vertebrate reservoir hosts. Borrelia burgdorferi spirochetes then multiply rapidly in feeding larvae and during the first days postrepletion. The number of spirochetes are then dramatically reduced during subsequent molting (6). Spirochetes persisting in the nymphal midgut upregulate OspA (7) and stay attached to the TROSPA receptor on the surface of the midgut epithelial cells (8). Spirochetes remain in this intimate relationship until the next blood meal. As the infected nymphs start feeding on the second host, *Borrelia* spirochetes sense appropriate physiochemical stimuli that trigger their replication (7, 9). Their numbers increase exponentially (10, 11), and the spirochetes downregulate OspA and upregulate OspC (7, 12). Simultaneously, ticks downregulate the production of TROSPA (8). These changes help spirochetes to detach from the midgut, penetrate into the hemolymph, migrate to the salivary glands (8), and infect the vertebrate host.

Understanding of Lyme borreliosis in Europe lags far behind that in the United States, mainly because the situation is complicated by the existence of several different species in the B. burgdorferi sensu lato complex that act as causative agents of the disease. To date, only a few papers regarding transmission of B. burgdorferi sensu lato strains by I. ricinus ticks have been published. Available publications suggest that the transmission of European *Borrelia* strains differs from the model cycle described for B. burgdorferi/I. scapularis (5, 13–15).

In this study, we present an updated view on the B. afzelii transmission cycle. We have performed a quantitative tracking of B. afzelii from infected mice to I. ricinus and back to naive mice. We further tested the role of tick saliva in infectivity and survival of B. afzelii spirochetes.

## RESULTS

### Borrelia afzelii-Ixodes ricinus transmission model.

**FIG 1:**
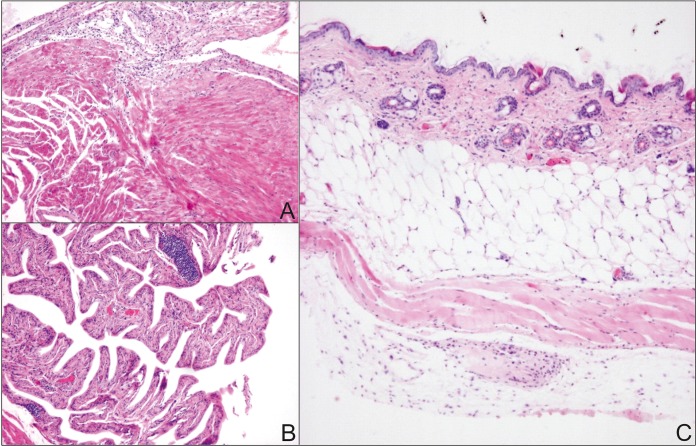
Pathological changes in target tissues of mice with B. afzelii infection. (A) Low-power section shows lymphocytic infiltration in the subepicardial space. (B) Urinary bladder mucosa shows lymphoid infiltration, including dense lymphoid aggregates within the submucosa. (C) Lymphocytic infiltrates are not prominent in the skin, and the majority of lymphoid infiltration is located deep in the connective tissue.

In order to understand the Lyme disease problem in Europe, the development of a transmission model is essential for the European vector I. ricinus and local *Borrelia* strains of the B. burgdorferi sensu lato complex. For this purpose, we established a reliable and robust transmission model employing C3H/HeN mice, I. ricinus ticks, and the B. afzelii CB43 strain isolated from local ticks (16). This strain develops systemic infections in mice and causes pathological changes in target tissues. Variably intensive lymphocytic infiltrations were detected in the heart, where the majority of inflammatory cells were concentrated in the subepicardial space with infiltration of myocytes (Fig. 1A). Inflammatory infiltration was prominent within the urinary bladder. The most prominent changes were in the submucosa, close to the basal membrane (Fig. 1B). In the skin, weak infiltration of the epidermis and dermis was documented; however, most lymphocytes were found in deep soft tissues (Fig. 1C). Borrelia afzelii CB43 also turned out to be highly infectious for I. ricinus ticks, as positive infection was detected in 90% to 100% of molted nymphs that fed on infected mice as larvae.

### B. afzelii population grows rapidly in engorged I. ricinus larvae and during molting to nymphs.

**FIG 2:**
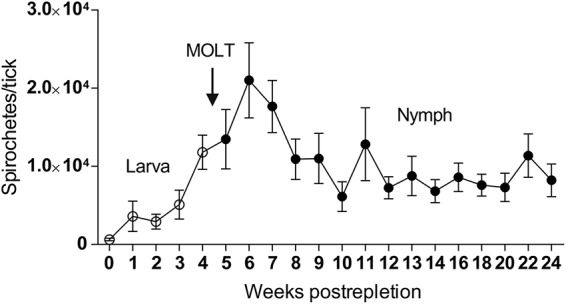
B. afzelii growth kinetics during larval-to-nymphal development in I. ricinus. Spirochetes multiply in engorged larvae as well as during molting to nymphs. Spirochetal proliferation then stops and spirochete numbers stay relatively stable from the 4th to 20th week postmolt. Each data point represents a mean from 20 individually analyzed ticks, and bars indicate SEM.

Studies on the dynamic relationship between the Lyme disease spirochete and its tick vector were previously performed on an I. scapularis/B. burgdorferi model (6, 10). Nevertheless, little is known about the growth kinetics of European B. afzelii in I. ricinus ticks. The number of spirochetes was determined in engorged I. ricinus larvae fed on B. afzelii-infected mice and then at weekly intervals until larvae molted to nymphs. Measurements were completed at the 20th week postmolt. The mean number of spirochetes in fully fed I. ricinus larvae examined immediately after repletion was relatively low, 618 ± 158 (± standard errors of the means [SEM]) spirochetes per tick. The spirochetes then multiplied rapidly in engorged larvae, and their numbers continued to increase during molting to nymphs. The maximum number of spirochetes, 21,005 ± 4,805 per tick, was detected in nymphs in the 2nd week after molting. Spirochetal proliferation then halted and the average spirochete number became relatively stable from the 4th to 20th week postmolt, slightly oscillating around the average number of about 10,000 spirochetes per tick (Fig. 2).

### B. afzelii numbers in I. ricinus nymphs dramatically drop during feeding.

**FIG 3:**
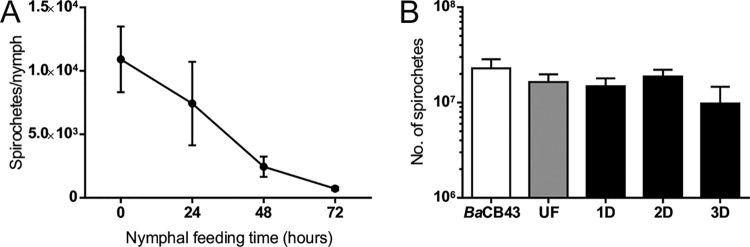
B. afzelii kinetics during nymphal feeding. (A) During feeding, spirochete numbers continuously decrease, from 10^4^ spirochetes/tick at the beginning to several hundred spirochetes/tick at the end of nymphal feeding. Each data point represents the mean from 20 individually analyzed ticks, and bars indicate SEM. (B) Spike-in control experiment revealed that the apparent drop in spirochete numbers during nymph feeding does not coincide with increased blood volume or presence of inhibitory contaminants. *Ba*CB43, 2.3 × 10^7^ B. afzelii spirochetes (5 ticks/group); UF, unfed I. ricinus nymphs; 1D, I. ricinus nymphs fed for 1 day; 2D, I. ricinus nymphs fed for 2 days; 3D, I. ricinus nymphs fed for 3 days.

**FIG 4:**
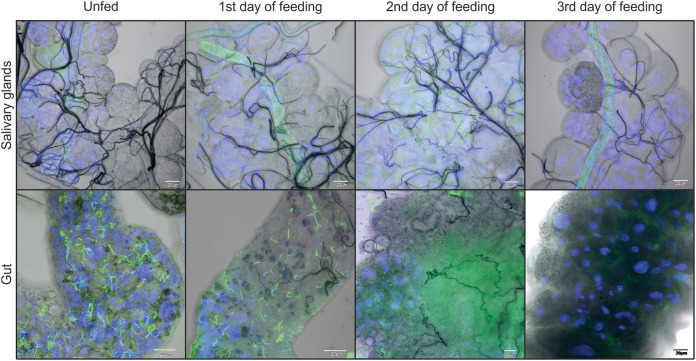
Presence of B. afzelii spirochetes in guts and salivary glands of feeding I. ricinus nymphs. Spirochetes are clearly visible in midguts of B. afzelii-infected nymphs. Their numbers significantly decrease during feeding. In contrast, spirochetes are not detectable in salivary glands of feeding I. ricinus nymphs. B. afzelii spirochetes are stained with anti-*Borrelia* antibody (green); nuclei are stained with DAPI (blue). Scale bars represent 20 μm.

We further examined the absolute numbers of B. afzelii spirochetes in infected I. ricinus nymphs during feeding. Nymphs were fed on mice and forcibly removed at time intervals of 24, 48, and 72 h after attachment, and the spirochetes were then quantified by quantitative PCR (qPCR). Prior to feeding, the mean number of spirochetes per nymph was 10,907 ± 2,590. After 24 h of tick feeding, the number of spirochetes decreased to 7,492 ± 3,294. In the following 2nd and 3rd day of blood intake, the numbers continued to drop to 2,447 ± 801 and 720 ± 138 spirochetes per tick, respectively (Fig. 3A). As this result was in striking contrast to the reported progressive proliferation of B. burgdorferi during I. scapularis nymphal feeding (10, 11), we confirmed the gradual decrease in B. afzelii spirochetes in the midguts of feeding I. ricinus nymphs using confocal immunofluorescence microscopy. In contrast, a parallel examination of the salivary glands from the same nymphs demonstrated that no spirochetes were detected in this tissue at any stage of feeding (Fig. 4).

### Ability of B. afzelii spirochetes to develop a persistent infection in mice increases with feeding time.

It is generally known that the risk of acquiring Lyme disease increases with the length of tick feeding (5). In subsequent experiments, we focused on the infectivity of B. afzelii transmitted via I. ricinus nymphs. To determine the minimum length of tick attachment time required to establish a permanent infection in mice, B. afzelii-infected nymphs were allowed to feed on mice for 24, 48, and 72 h (10 nymphs per mouse). Mouse infection was assessed in ear biopsy specimens 3 weeks after tick removal. The ability of B. afzelii spirochetes to promote a persistent infection increased with the length of tick attachment. All mice exposed to the bite of B. afzelii-infected ticks for 24 h remained uninfected, whereas 8/10 mice exposed for 48 h and 10/10 mice exposed for 72 h became infected. These results show that the time interval between 24 and 48 h of exposure to the B. afzelii-infected tick is critical for the development of a systemic murine infection.

### B. afzelii spirochetes are already present in the murine dermis on the first day of tick feeding.

**FIG 5:**
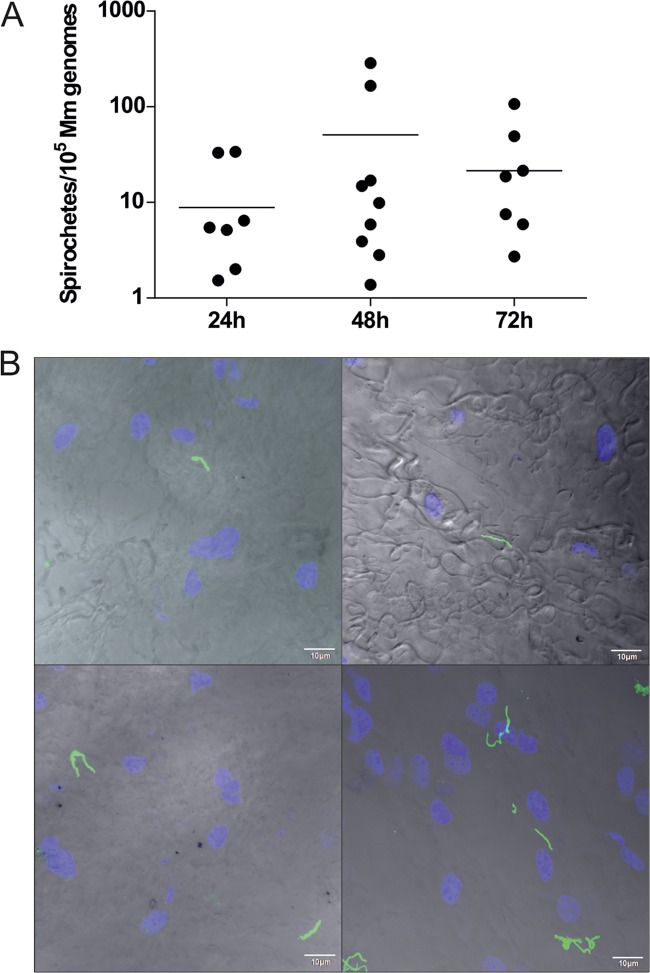
Timing of B. afzelii transmission from I. ricinus nymph to mouse. Skin biopsy specimens from mice exposed to infected ticks for various time periods were tested for infection by qPCR (A) or confocal microscopy (B). B. afzelii spirochetes are present in the skin during the early stages of tick feeding. (A) Each data point represents the number of B. afzelii spirochetes/10^5^ murine genomes in individually analyzed skin biopsy specimens. (B) Presence of B. afzelii spirochetes in murine skin at the 24-h time point. B. afzelii spirochetes are stained with anti-*Borrelia* antibody (green); nuclei are stained with DAPI (blue).

The delay in development of a B. afzelii infection in mice may support the notion that the spirochetes are still travelling toward the tick salivary glands during the first day after attachment. To test this hypothesis, we determined the number of B. afzelii organisms in murine skin biopsy specimens from the tick feeding site at time intervals of 24, 48, and 72 h after feeding. Skin biopsy specimens from 9/10, 10/10, and 10/10 mice were PCR positive at time intervals of 24, 48, and 72 h, respectively. Analysis by qPCR further revealed that there were no significant differences in the number of spirochetes in skin samples at defined time intervals (Fig. 5A). This result was also confirmed by confocal microscopy, revealing clearly the presence of spirochetes in murine skin biopsy specimens during the first day of tick feeding (Fig. 5B). Together with the rapid decrease in spirochetal number in nymphal midguts during feeding (Fig. 3A and 4), these results imply that the migration of spirochetes to the host commences soon after the blood meal uptake.

### Tick saliva does not protect the early B. afzelii spirochetes against host immunity.

**FIG 6:**
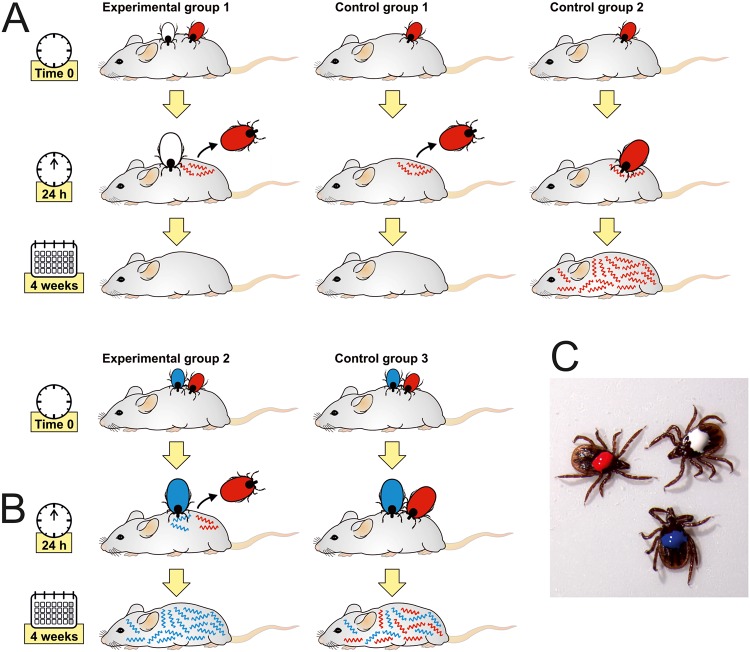
Role of tick saliva in B. afzelii survival. Presence of neither uninfected ticks (A) nor B. burgdorferi-infected ticks (B) and their saliva is sufficient for protection of early B. afzelii against their elimination by the host immune system. (C) Differentially labeled I. ricinus nymphs. White, uninfected nymph; red, B. afzelii-infected nymph; blue, B. burgdorferi-infected nymph.

The apparent contradiction between the early entry of B. afzelii spirochetes into the vertebrate host and their delayed capability to develop a permanent infection supports the concept of the tick saliva’s role in the successful dissemination and survival of spirochetes within the host body. In order to verify that tick saliva is essential for B. afzelii survival in mice, we designed and performed the following experiment. In experimental group 1, uninfected I. ricinus nymphs (white labeled) were allowed to feed simultaneously with B. afzelii-infected nymphs (red labeled) at the same feeding site. After 24 h of cofeeding, B. afzelii-infected nymphs were removed, while uninfected ticks were fed on mice until repletion and served as a source of saliva. In control group 1, B. afzelii-infected nymphs fed for 24 h without any support of uninfected ticks. In control group 2, B. afzelii-infected nymphs were allowed to feed until repletion. Four weeks later, B. afzelii infections in ear, heart, and urinary bladder biopsy specimens were examined by PCR. No infection was detected in any of the examined tissues in experimental and control group 1, where the infected ticks fed for only 24 h. In contrast, all tissues were PCR positive in control group 2, where the infected nymphs fed until repletion (Fig. 6A). These results revealed that the presence of uninfected ticks and their saliva is not sufficient to protect early spirochetes against elimination by the host immune system.

A possible explanation of this unanticipated result is that unlike those of the uninfected tick, the salivary glands of *Borrelia*-infected ticks express a different spectrum of molecules that assist their transmission and survival within the vertebrate host (17–19). Therefore, we also examined the protective effect of saliva from *Borrelia*-infected nymphs. The experimental setup was the same as that described above, with one exception: in experimental group 2, nymphs infected with a different strain of B. burgdorferi were allowed to feed until repletion next to B. afzelii-infected nymphs that were removed after 24 h. In control group 3, B. afzelii-infected and B. burgdorferi-infected nymphs were allowed to feed until repletion. Four weeks after repletion, mice were specifically examined for the presence of one or both *Borrelia* strains using *rrs-rrlA* intergenic spacer (IGS) PCR amplification. All mice in experimental group 2 were positive for B. burgdorferi, while B. afzelii was not detected in any of the analyzed murine tissues. All mice in control group 3 tested positive for both B. afzelii and B. burgdorferi (Fig. 6B). This result implies that the saliva from B. burgdorferi-infected ticks also was not capable of ensuring survival of B. afzelii transmitted to mice at the early feeding stage.

### Infectivity by B. afzelii is gained in the midgut and changes during nymphal feeding.

Another possible explanation for the delayed capability of B. afzelii to infect mice was that infectivity of the spirochetes changed during the course of nymphal feeding. To test the infectivity of B. afzelii during different phases of nymphal feeding, B. afzelii-containing guts were dissected from unfed I. ricinus nymphs and nymphs fed for 24 h, 48 h, and 72 h and subsequently injected into C3H/HeN mice (5 guts/mouse). B. afzelii spirochetes from unfed nymphs were not infectious for mice. Spirochetes from nymphs fed for 24 h infected 3 out of 5 inoculated mice, and all mice became infected after the injection of spirochetes from nymphs fed for 48 h. Interestingly, only 1 out of 5 mice inoculated with spirochetes from nymphs fed for 72 h established B. afzelii infection. This result suggests that the capability of B. afzelii spirochetes to infect mice is gained in the tick gut and peaks at about the 2nd day of feeding.

### Infectivity of B. afzelii is linked to differential gene expression during tick feeding and transmission.

**FIG 7:**
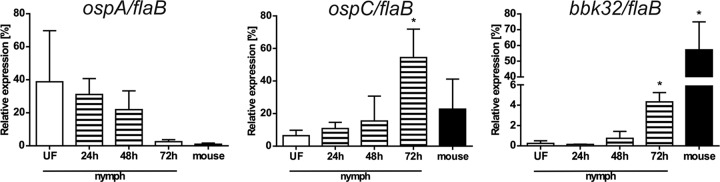
Comparative analysis of *ospA*, *ospC*, and *bbk32* gene expression in B. afzelii spirochetes during tick feeding and mouse infection. Each data point represents the mean from 3 individually analyzed samples, and bars indicate SEM. *, *P* < 0.05.

Previous research demonstrated that transmission of B. burgdorferi from I. scapularis to the host is associated with changes in expression of genes encoding outer surface proteins OspA and OspC or the fibronectin-binding protein BBK32 (7, 20–22). In order to examine whether the infectivity of B. afzelii depends on expression of orthologous genes, we performed qPCR analysis to determine the status of *ospA*, *ospC*, and *bbk32* expression by B. afzelii spirochetes in unfed and feeding I. ricinus nymphs as well as in murine tissues 4 weeks postinfection. The gene encoding OspA was abundantly expressed in unfed ticks, downregulated during tick feeding, and hardly detectable in mice. The B. afzelii *ospC* gene was weakly expressed in unfed I. ricinus nymphs. Its expression steadily increased during feeding, with the highest levels of *ospC* mRNA at the 3rd day of feeding. Significant *ospC* expression was also detected in mice with a permanent B. afzelii infection. Similarly, a gradual upregulation of *bbk*32 was evident with the progress of tick feeding, and gene transcription was fully induced during mammalian infection (Fig. 7).

## DISCUSSION

Understanding the dynamics of *Borrelia* spirochete transmission is crucial for development of strategies for preventing Lyme disease. Recently, we managed to implement a reliable transmission model for European Lyme disease that involves the vector I. ricinus and the most common causative agent of borreliosis in Europe, B. afzelii spirochetes. This allowed us to quantitatively track the growth kinetics and infectivity of B. afzelii during the I. ricinus life cycle and compare the results to data known for the I. scapularis/B. burgdorferi model.

In nature, infection is acquired by larval or nymphal ticks feeding on an infected host. Absolute quantification of B. afzelii spirochetes during larval development and molting to nymphs revealed that I. ricinus larvae imbibe relatively low spirochete numbers (∼600 per tick). The number of B. afzelii organisms then gradually increases during larval molting and reaches its maximum of about 20,000 spirochetes per tick 2 weeks after molting to nymphs. The level then stabilizes at about 10,000 spirochetes in starving nymphs (Fig. 2). This course of spirochetal burden is roughly in line with the data reported for I. scapularis/B. burgdorferi (6). However, compared to our observations, these authors described a dramatic decrease in B. burgdorferi numbers during I. scapularis molting. They speculated that it was due to depleted amounts of *N*-acetylglucosamine, an important building block of integumentary chitin but also a key component for spirochetal development. The limited availability of other nutrients might also be the reason for halted proliferation of spirochetes in molted nymphs. With its adoption of a parasitic lifestyle, the bacterium is an auxotroph for all amino acids, nucleotides, and fatty acids. It also lacks genes encoding enzymes for the tricarboxylic acid cycle and oxidative phosphorylation (23, 24). Therefore, *Borrelia* spirochetes in the tick midgut are completely dependent on nutrients derived from ingested blood.

A striking difference between I. ricinus/B. afzelii and I. scapularis/B. burgdorferi was observed in spirochete numbers in the nymphal midgut during feeding. We found that B. afzelii numbers dramatically decrease from about ∼10,000 spirochetes present in flat I. ricinus nymphs to only ∼700 spirochetes in nymphs fed for 3 days (Fig. 3A). This result is in sharp contrast with the data previously published for I. scapularis/B. burgdorferi. Using antibody-based detection, De Silva and Fikrig demonstrated that the total number of B. burgdorferi organisms increased from several hundred in starved nymphs to almost 170,000 spirochetes on the 3rd day of nymphal feeding (10). Later, these data were confirmed in a qPCR study showing that B. burgdorferi spirochetes in tick midguts increased 6-fold, from about 1,000 before attachment to about 6,000 at 48 h after attachment (11). The observation that numbers of spirochetes in ticks decrease as nymphs acquire their blood meal is intriguing, especially as it goes against what has been observed previously. The apparent drop in numbers during nymph feeding coincides with increased blood volume. Moreover, not all DNA extraction methods remove inhibitory contaminants. To test whether the qPCR can be inhibited by blood components or influenced by increased blood volume, we performed a spike-in control experiment which revealed that spirochetal decrease during nymphal feeding is due to the transmission of spirochetes and not inhibition or increased blood volume (Fig. 3B).

It is commonly known that the risk of Lyme disease increases with the length of time a tick is attached. It was stated that I. scapularis ticks infected with B. burgdorferi removed during the first 2 days of attachment do not transmit the infection (11, 20). Our data show that B. afzelii spirochetes require less time to establish a permanent infection. Most mice became infected by 48 h of attachment. This is in agreement with the previously published results showing that B. afzelii-infected I. ricinus nymphs transmit the infection earlier than B. burgdorferi-infected ticks (13).

Nevertheless, quantification by qPCR as well as microscopic examination of B. afzelii in the mouse dermis revealed that B. afzelii spirochetes enter the host earlier than they are able to develop a systemic infection (Fig. 5). This is in agreement with their significant decrease in the tick midgut during feeding (Fig. 3A and 4) and suggests that B. afzelii spirochetes leave the nymphs as early as the first day of feeding. The presence of spirochetes in mouse dermis prior to becoming infectious was also reported for I. scapularis/B. burgdorferi. Ohnishi et al. observed noninfectious spirochetes in skin samples from mice that were exposed to B. burgdorferi-infected I. scapularis nymphs for less than 53 h (20). Moreover, Hodzic et al. also reported the presence of B. burgdorferi spirochetes in four out of eight mice 24 h after I. scapularis attachment (25). These data suggest that *Borrelia* spirochetes invade the host at very early time points of tick feeding, but early spirochetes are not able to develop a systemic infection. There could be two explanations for this observation. First, bioactive molecules present in tick saliva are crucial for successful dissemination and survival of spirochetes within the host body. Therefore, the early spirochetes cannot colonize the host without sufficient protection and support of the tick saliva (26, 27). Second, early spirochetes that are transmitted to the vertebrate host are not infectious. A substantial body of work has been performed to elucidate the various tick bioactive molecules, mainly comprising a complex cocktail of salivary proteins that dampens the host’s defenses against blood loss and the development of inflammatory and complement reactions at the feeding site (28). Several tick molecules have been suggested to be crucial for *Borrelia* acquisition in ticks and transmission to the next host during subsequent feeding (reviewed in reference 29). To test the role of tick saliva in survival of early spirochetes, we performed a cofeeding experiment in which the early B. afzelii spirochetes were under the protection of uninfected ticks or ticks infected with B. burgdorferi (Fig. 6). This experiment clearly showed that the presence of tick saliva is not sufficient for protection and survival of early spirochetes, as all mice remained uninfected with B. afzelii spirochetes. Therefore, we tested how infectivity of B. afzelii changes during tick feeding. A number of studies provide solid evidence that *Borrelia* spirochetes change expression of their surface antigens during feeding and transmission to the host, making it possible for spirochetes to specifically adapt to the tick or the host environment as required (7, 30, 31). Changes in gene expression of our model spirochete seem to be the main event that promotes increasing infectivity during tick feeding. Borrelia afzelii spirochetes in unfed ticks showed high levels of expression of *ospA* and negligible expression of *ospC* and *bbk32*. In this tick model, spirochetes were not infectious for mice. As feeding progressed, *ospA* was downregulated and *ospC* and *bbk32* were upregulated, which correlated with increasing infectivity of B. afzelii. The highest level of infection was observed in mice inoculated with spirochetes from nymphs fed for 48 h. By this time, all mice had developed the infection. Interestingly, spirochetes from nymphs fed for 72 h infected only one out of five mice. This decrease is likely associated with a concomitant, substantially reduced number of B. afzelii organisms in the midguts of nymphs fed for 3 days (Fig. 3A and 4). Similar findings also were reported for B. burgdorferi. It was demonstrated that viable B. burgdorferi organisms in unfed I. scapularis nymphs are highly attenuated in their ability to infect mice relative to spirochetes obtained from recently fed ticks. This finding suggests that tick feeding induces critical changes that specifically prepare the spirochete for infection of the mammalian host (32).

The route of *Borrelia* spirochete transmission has been broadly discussed since its discovery. In 1984, Burgdorfer suggested that spirochetal development in most ticks (I. scapularis and I. ricinus) occurs in the midgut. Additional tissues, including salivary glands, were considered to be free of spirochetes in most of the ticks. It was suggested that transmission occurs by regurgitation of infected gut contents or via saliva by ticks with a generalized infection (4). Benach et al. presented similar findings in their extensive histological study. They stated that B. burgdorferi organisms are able to enter the hemocoel during the midfeeding period and develop a systemic infection in the hemolymph and central ganglion. However, B. burgdorferi organisms were never seen within the lumen of the salivary gland or attached to cells of the salivary acini (2). The salivary route of Lyme disease transmission came into consideration in 1987, when Ribeiro and colleagues reported the presence of spirochetes in saliva of pilocarpine-treated ticks (3), and then was broadly accepted after microscopic detection of spirochetes within the salivary glands and ducts of fully fed I. scapularis nymphs (33). Nevertheless, the spirochete numbers present in salivary glands of I. scapularis nymphs are minuscule and hardly detectable (34, 35).

In our study, we were not able to detect B. afzelii spirochetes in the salivary glands at any stage of tick feeding. The absence of spirochetes in salivary glands is surprising, since large numbers of spirochetes were supposed to pass from the midgut to the feeding lesion during the three-day course of nymphal feeding. A possible explanation is that the gland-associated spirochetes were not detectable by the chosen method or that these findings raise the possibility of an alternative route of B. afzelii transmission. We suggest that active reverse migration of motile B. afzelii spirochetes from the midgut to the mouthpart should be further tested as a possible alternative to the traditional salivary transmission route. The idea of B. afzelii transmission avoiding I. ricinus hemocoel and salivary glands also is indirectly supported by our recent research showing that silencing of tick immune molecules or elimination of phagocytosis in tick hemocoel by injection of latex beads had no obvious impact on B. afzelii transmission (36–38).

From our results, we propose the following mechanism of B. afzelii transmission. Borrelia afzelii in flat I. ricinus nymphs represents a relatively abundant population of spirochetes. Once the tick finds a host, B. afzelii organisms immediately start their transmission to the host. B. afzelii also seems to be less dependent on its tick vector. The main requirement for successful host colonization is the change in outer surface protein expression that occurs in the tick gut during the course of feeding. Spirochetes switched to the proper, vertebrate mode are then able to survive within the host even if the tick is not present. The 24- to 48-h time window between tick attachment and transmission of infectious spirochetes is the critical period in the whole process. Our findings suggest that salivary delivery as well as alternative transmission routes should be tested in future studies as possible mechanisms of transmission of different *Borrelia* species. Better understanding of the transmission cycles forms a basis for preventive and therapeutic strategies against Lyme disease.

## MATERIALS AND METHODS

### Laboratory animals.

Ixodes ricinus larvae and nymphs were obtained from the breeding facility of the Institute of Parasitology, Biology Centre, Czech Academy of Sciences. Ticks were maintained in wet chambers with a humidity of about 95%, temperature of 24°C, and day/night period set to 15/9 h. To prepare both infected and uninfected I. ricinus nymphs, the larvae were fed on either infected or uninfected mice and allowed to molt to nymphs, and after 4 to 6 weeks they were used for further experiments. Inbred, pathogen-free C3H/HeN mice (The Jackson Laboratory, Bar Harbor, ME) were used for the pathogen transmission experiments.

### Ethics statement.

All experimental animals were treated in accordance with the Animal Protection Law of the Czech Republic, no. 246/1992 Sb., ethics approval no. 161/2011. The animal experimental protocol was approved by the Czech Academy of Sciences Animal Care and Use Committee (protocol permit number 102/2016).

### Infection of mice and ticks.

Low-passage strains of B. afzelii CB43 (16) and B. burgdorferi SLV-2 (39) were grown in Barbour-Stonner-Kelly H (BSK-H) medium (Sigma-Aldrich, St. Louis, MO, USA) at 33°C for 5 to 7 days. Six-week-old female C3H/HeN mice were infected by subcutaneous injections of 10^5^ spirochetes per mouse. The presence of spirochetes in ear biopsy specimens was verified 3 weeks postinjection by PCR. Clean I. ricinus larvae were fed on infected mice until repletion and allowed to molt. Nymphs were considered to be infected if >90% of them were PCR positive.

### Nucleic acid isolation and cDNA preparation.

DNA was isolated from individual larvae, nymphs, and murine tissues (ear, skin, heart, and urinary bladder) using a NucleoSpin tissue kit (Macherey-Nagel, Düren, Germany) according to the manufacturer’s protocol.

Total RNA was extracted from nymphs and murine tissues (ear and urinary bladder) using a NucleoSpin RNA kit (Macherey-Nagel) according to the manufacturer’s protocol. Isolated RNA (1 μg) served as a template for reverse transcription into cDNA using a Transcriptor high-fidelity cDNA synthesis kit (Roche, Basel, Switzerland). All cDNA preparations were prepared in biological triplicates.

### PCR.

Detection of spirochetes in ticks, as well as in murine tissues, was performed by nested PCR amplification of a 222-bp fragment of a 23S rRNA gene (40). PCR contained 12.5 μl of FastStart PCR master mix (Roche), 10 pmol of each primer, template (4 μl of DNA for the first round, 1-μl aliquot of the first PCR product in the second round), and PCR water up to 25 μl. Primers and annealing temperatures are listed in Table 1.

**TABLE 1:** Primers and probes used in this study

Organism	Gene	Primer name	Sequence 5′→3ʹ	Annealing temp (°C)	Product size (bp)	Reference or source
*Borrelia* spp.	23S rRNA	Bor-1	AGAAGTGCTGGAGTCGA	53	260	40
		Bor-2	TAGTGCTCTACCTCTATTAA			
		Bor-3	GCGAAAGCGAGTCTTAAAAGG	58	222	
		Bor-4	ACTAAAATAAGGCTGAACTTAAAT			
*Borrelia* spp.	*rrs-rrlA* IGS	Bb IGS-F	GTATGTTTAGTGAGGGGGGTG	56	Different for different species	41
		Bb IGS-R	GGATCATAGCTCAGGTGGTTAG			
		Bb IGS-Fn	AGGGGGGTGAAGTCGTAACAAG	60		
		Bb IGS-Rn	GTCTGATAAACCTGAGGTCGGA			
*Borrelia* spp.	Flagellin	FlaF1A	AGCAAATTTAGGTGCTTTCCAA	60	154	42
		FlaR1	GCAATCATTGCCATTGCAGA			
		Fla Probe1	TGCTACAACCTCATCTGTCATTGTAGCATCTTTTATTTG			
Mus musculus	*actin*	Mmact-F	AGAGGGAAATCGTGCGTGAC	60	137	43
		Mmact-R	CAATAGTGATGACCTGGCCGT			
		Mmact-P	CACTGCCGCATCCTCTTCCTCCC			
Borrelia afzelii	*ospA*	RTospA-F	GGTTCTGGAGTGCTTGAAGG	55	112	45
		RTospA-R	TGTTTTGCCATCTTCTTTG			
Borrelia afzelii	*bbk32*	RTbbk32-F	CACGTCTTGACAACCTTGCT	55	117	
		RTbbk32-R	CCTTGCACTCACTTGAATATAG			
Borrelia afzelii	*flaB*	RTflaB-F	GTTCATGTGGGAGCAAATCA	55	120	
		RTflaB-R	ACCCTCTTGAACAGGTGCAG			
Borrelia afzelii	*ospC*	BaospC-F	GCAGGAGCCTATGCAATATCA	60	150	This study
		BaospC-R	TTTGCCAAGATCTGCATGAC			

Differentiation of B. afzelii and B. burgdorferi strains was performed by nested PCR amplifying a part of the *rrs-rrlA* IGS region (41). Reaction conditions were the same as those described above, and primers and annealing temperatures are listed in Table 1.

### qPCR.

Total spirochete load was determined in murine and tick DNA samples by quantitative real-time PCR (qPCR) using a LightCycler 480 (Roche). The reaction mixture contained 12.5 μl of FastStart universal probe master (Rox) (Roche), 10 pmol of primers FlaF1A and FlaR1, 5 pmol of TaqMan probe Fla Probe1 (42) (Table 1), 5 μl of DNA, and PCR water up to 25 μl. The amplification program consisted of denaturation at 95°C for 10 min, followed by 50 cycles of denaturation at 95°C for 15 s and annealing plus elongation at 60°C for 1 min.

Quantification of murine β-*actin* was performed using MmAct-F and MmAct-R primers and a MmAct-P TaqMan probe (43) (Table 1). Reaction and amplification conditions were the same as those described above. The spirochete burden in murine tissues was expressed as the number of spirochetes per 10^5^ murine β-*actin* copies. The spirochete burden in ticks was calculated as the total number of spirochetes in the whole tick body.

cDNAs from B. afzelii-infected I. ricinus nymphs as well as murine tissues served as templates for quantitative expression analyses by relative qPCR. The reaction mixture contained 12.5 μl of FastStart universal SYBR green master, Rox (Roche), 10 pmol of each primer (Table 1), 5 μl of cDNA, and PCR water up to 25 μl. The amplification program consisted of denaturation at 95°C for 10 min, followed by 50 cycles of denaturation at 95°C for 10 s, annealing at 60°C for 10 s, and elongation at 72°C for 10 s. Relative expression of *ospA*, *ospC*, and *bbk32* was normalized to that of *flaB* using the ΔΔ*C_T_* method (44).

### Spike-in experiment.

To test whether the qPCR can be inhibited by blood components or influenced by increased blood volume, a spike-in control experiment was performed. Homogenates from unfed clean nymphs and nymphs fed for 24, 48, and 72 h (5 ticks/group) were spiked with defined amounts of B. afzelii spirochetes (2.3 × 10^7^ spirochetes/homogenate). DNA from all homogenates then was isolated, and spirochete loads were quantified using methods described above.

### Preparation of murine and tick tissues for confocal microscopy.

Borrelia afzelii-infected I. ricinus nymphs were fed on mice for 24 h. Skin biopsy specimens from the tick feeding site then were dissected. Guts and salivary glands of unfed nymphs and nymphs fed for 24 h or 48 h or fully fed and infected with B. afzelii were dissected in phosphate buffer (30 nymphs/time point). Dissected tissues were immersed in 4% paraformaldehyde for 4 h at room temperature. Tissues were then washed three times for 20 min each time in phosphate-buffered saline (PBS) and permeabilized with 1% Triton X-100 (Tx) in PBS containing 1% bovine serum albumin (Sigma) at 4°C overnight. The next day, *Borrelia* spirochetes in tissues were stained with primary rabbit anti-B. burgdorferi antibody (1:200; Thermo Fisher Scientific) in PBS-Tx (0.1% Tx in PBS) for 4 h at room temperature. Tissues were then washed three times for 20 min each time in PBS-Tx and stained with Alexa Fluor 488 goat anti-rabbit secondary antibody (Life Technologies, Camarillo, CA, USA), 1:500 in PBS-Tx, for 2 h at room temperature. Tissues were counterstained with 4′,6-diamidino-2-phenylindole (DAPI) for 10 min and washed two times for 10 min each time in PBS. Slides then were mounted in DABCO and examined using an Olympus FluoView FV1000 confocal microscope (Olympus, Tokyo, Japan). Whole salivary glands were thoroughly scanned for the presence of spirochetes (12 to 20 fields of view per salivary gland).

### Preparation of murine tissues for histology.

*Borrelia afzelii*-infected or clean I. ricinus nymphs were fed on mice until repletion (5 mice/group, 10 nymphs/mouse). Four weeks later, murine tissues (skin, heart, and urinary bladder) from B. afzelii-infected and uninfected mice were fixed in 10% buffered formalin and embedded in paraffin using routine procedures. Three-μm thin sections were cut and stained with hematoxylin and eosin. Slides were examined using an Olympus BX40 light microscope (Olympus).

### Needle inoculation of infected tick midguts.

B. afzelii-containing guts from unfed I. ricinus nymphs and nymphs fed for 24 h, 48 h, and 72 h were dissected and suspended in BSK-H medium (Sigma). Guts were subsequently injected into C3H/HeN mice (5 guts/mouse in a 200-μl volume, 5 mice/time point).

### Statistical analysis.

Data were analyzed by GraphPad Prism 6 for Windows, version 6.04, and an unpaired Student's *t* test was used for evaluation of statistical significance. A *P* value of <0.05 was considered statistically significant. Error bars in the graphs show the standard errors of the means.
